# A Review of the Role of Micronutrients and Bioactive Compounds on Immune System Supporting to Fight against the COVID-19 Disease

**DOI:** 10.3390/foods10051088

**Published:** 2021-05-14

**Authors:** Montaña Cámara, María Cortes Sánchez-Mata, Virginia Fernández-Ruiz, Rosa María Cámara, Elena Cebadera, Laura Domínguez

**Affiliations:** Nutrition and Food Science Department, Pharmacy Faculty, Complutense University of Madrid (UCM), Plaza Ramón y Cajal, s/n, E-28040 Madrid, Spain; cortesm@farm.ucm.es (M.C.S.-M.); vfernand@farm.ucm.es (V.F.-R.); rosacama@ucm.es (R.M.C.); ecebadera@farm.ucm.es (E.C.); ladoming@ucm.es (L.D.)

**Keywords:** health claims, immune system, micronutrients, vitamin D, food supplements, COVID-19

## Abstract

Micronutrients are critical for an adequate function of the immune system and play a vital role in promoting health and nutritional well-being. The present work is aimed at reviewing (1) the role of micronutrients in helping the immune system to fight against the COVID-19 disease through the diet with food or food supplements and (2) the potential use of food health claims regarding immune function according to the European Food Safety Authority (EFSA) requirements. Till date, there are some health claims authorized by the European Commission that refer to the role of certain essential nutrients (vitamins B_6_, B_9_, B_12_, A, D, C, and Cu, Fe, Se) to contribute to the proper functioning of the immune system. Vitamins D, C, Zn, and Se, have been thoroughly studied as a strategy to improve the immune system to fight against COVID-19 disease. From all the micronutrients, Vitamin D is the one with more scientific evidence suggesting positive effects against COVID-19 disease as it is linked to a reduction of infection rates, as well as an improved outcomes in patients. To validate scientific evidence, different clinical trials are ongoing currently, with promising preliminary results although inconclusive yet.

## 1. Introduction

Infectious diseases have been one of the main causes of human death since its origin. In order to protect the organism from infections, the immune system is able to identify and eliminate pathogens or microorganisms as bacteria, viruses, fungi, or parasites, through different mechanisms, including innate immunity (neutrophils and macrophages) and the acquired immunity. The immune system involves various blood-borne factors as the complement, antibodies and cytokines, along with different types of cells [1,2]. In addition, the evolutionary capacity of pathogens to create ways to avoid detection by the immune system, thus being able to infect the host or host organism [3,4,5]. The evolution of an infectious disease in an individual involves a sequence of interactions between the pathogen and the host, including the invasion, development, and colonization of the host by the microorganism, as well as the immune response of the host. The result of this confrontation will lead to disease or resistance.

Epidemics and pandemics, such as smallpox, measles, and influenza, amongst others, have decimated world population to limits that would be unthinkable to us today, since the only tool that humans had was their own immune system. The situation took a radical turn from the XIX-XX centuries, due to the discovery of vaccines and antibiotics, along with improved hygiene, health, and food conditions. Nowadays, one of the challenges for Immunology in the XXI century is undoubtedly the suppression of infectious diseases on a global scale, such as avian influenza, AIDS, malaria, or COVID-19 [6]. In many cases, the difficulty in finding an effective remedy is due to (1) individual immunodeficiency situation and (2) the appearance of viral mutants that escape from immune response and resistant pathogens; where the treatment does not have the desired effect.

It is undoubtable that to provide protection against infectious diseases, public hygiene practices and vaccination are the most effective measures [7]. However, the level of protection of vaccines is variable, and they are not available against all viruses. The new generation of vaccines developed by genetic engineering allows us to eliminate the virulent genes of an infectious agent without altering the ability to stimulate an immune response of the individual in which it is inoculated. Although large-scale vaccination has difficulties as a consequence of high costs of vaccines—especially those of the latest generation—, their distribution in remote and difficult-to-access places

COVID-19 disease is characterized by a long and silent incubation period that is mostly asymptomatic but contagious, allowing a rapid spread across the world provoking severe acute respiratory syndrome, the main cause of morbidity and mortality [6,7,8]. Although it presents different symptoms depending on the group of population, it is observed that those individuals with a compromised immune system may be at higher risk of suffering serious complications similar to what is seen with other respiratory diseases. Thus, to reduce the impact of respiratory and other infections, other actions to maintain and protect the immune system is needed.

There is an undeniable relationship between an adequate diet and good health, and that correct eating habits can contribute to the prevention of diseases of different types, including some chronic diseases, such as obesity, cardiovascular disease, or some types of tumors. In the case of infections, the first obstacle is its enormous complexity and the multiple factors involved, which makes difficult to demonstrate a clear effect of food components in immune mechanisms. Different nutrients have demonstrated to be fundamental for an efficient function of the immune system. These include essential amino acids, vitamins (A, B_6_, B_12_, C, E, folic acid), linoleic acid, minerals (Cu, Fe, Se, and Zn). It is proved that immunity is decreased by deficiencies in some of these nutrients [9]. Poor nutritional habits or other circumstances can cause deficiencies in the intake of certain nutrients with serious consequences for the development of COVID-19 [10,11,12,13,14]. Thus, boosting the immune system can be a good target for functional foods and food supplements development [15,16,17]. We also have to consider that interactions between nutrients may negatively impact the immune function [18]. Thus, more studies should be performed for more reliable recommendations [10,19].

The present work is aimed at reviewing (1) the role of micronutrients in helping the immune system to fight against the COVID-19 disease through the diet with food or food supplements, and (2) the potential use of food health claims regarding immune function according to the European Food Safety Authority (EFSA) requirements. To accomplish that goal, we first reviewed the Health Claims approved in the European Union (EU) related to the immune system stimulation; secondly, we described the main food sources of nutrients and bioactive compounds involved in immune system stimulation; and third, we performed a scientific literature search on the evidence of micronutrients and immune system health benefits against the COVID-19 disease.

## 2. Materials and Methods

The protocol used in this work includes the following steps:

*(1) Review of the International Health Claims approved in EU related to the immune system stimulation.* This review was performed using the public information included in the European Register of nutrition and health claims made on food and food supplements [20] according to the Regulation [21,22,23] No 1924/2006 and 1925/2006.

*(2) Scientific literature research.* We conducted a literature search on:

- PubMed Database [http://www.ncbi.nlm.Nih.gov/PubMed] (accessed on 6 February 2021).
  Inclusion criteria. English language, year of publication (last ten years), human studies and the following keywords “immune system”, “food”, and “food supplement”, “micronutrients”, as well as “COVID-19”.
  Exclusion criteria. Studies related to other health benefits different from immune system benefits were excluded.
- Clinical trials search: we used two databases, the World Health Organization’s International Clinical Trials Registry Platform [https://www.who.int/clinical-trials-registry-platform] [24] and on the website ClinicalTrials.gov, a resource provided by the NIH-US National Library of Medicine [https://clinicaltrials.gov/ct2/results?cond=COVID-19] [25] (accessed on 18 January 2021).
  Inclusion criteria. English language, year of publication (2020, 2021), human studies and the following keywords “COVID-19” and “vitamin” and/or “food supplement” and/or “micronutrients”.
  Exclusion criteria. Those trials focused on health benefits of food supplement consumption different from COVID-19, as well as studies relating the effect of several drugs in combination with “vitamin”, “food supplement”, or “micronutrients”.

## 3. Results

### 3.1. Health Claims Approved in EU Regarding Immune System Stimulation

An effective immune response requires an adequate host nutritional status. In 2011, EFSA Panel on Dietetic Products, Nutrition and Allergies (NDA Panel) provided a scientific opinion about the assessment and substantiation of health claims in relation to specific food (e.g., cranberry, blackcurrant, mangosteen fruit, shitake, maitake, etc.) or food constituents (e.g., glucosinolates, papain, bromelain, cryptoxanthin from orange juice, etc.) and the immune function or immune system, contribution to body defenses against external agent and stimulation of immunological responses [26]. The Panel concluded that the claimed effects were not sufficiently defined and that the stimulation of the immune system was not demonstrated to be a physiological effect per se. Therefore, a cause-and-effect relationship between the food or food constituents in question and the proposed health benefits cannot be established. The majority of the human intervention studies provided to support the approval of these health claims, did not meet the specific EFSA requirements because of the lack of appropriate clinical outcomes related to infections, inaccuracies related to the nature of the infectious disease, and the use of non-validated questionnaires to evaluate some important parameters of common colds (incidence, symptoms duration, etc.).

Lately, to help applicants, EFSA published in 2016 a Guidance including the required specifications and criteria to approve health claims related to the immune system [27]. Some valid markers include activities of specific cells (lymphocytes, phagocytes, killer cells, cytolytic T cells); synthesis of cellular mediators; concentrations of particular lymphoid populations and immunoglobulins, etc.

Regarding the specific health claim “defence against pathogens in the respiratory system”, human intervention studies are needed. Studies should show an effect on specific clinical outcome of respiratory infections (incidence, seriousness, symptoms duration, etc.) of both the upper and lower respiratory tract (rhinitis, sinusitis, common cold, as well as pneumonia, bronchitis, and bronchiolitis), to be considered a good scientific basis to substantiate the approval of health claim. Microbiological data, as well as clinical and differential diagnosis, of respiratory tract infections following well-defined criteria are also considered important factors to properly exclude non-infectious causes, such as allergies.

Till date, nutrients with authorized health claims related to the immune system function are the following (Table 1). According to Regulation (EC) 1924/2006 and (EU) 1169/2011, this positive effect would be achieved if the nutrient is consumed in sufficient quantities to cover the daily nutritional requirements; that is called a “significant amount”, and it is equivalent to 15% of the Nutrient Reference Values (NRV) for each specific case (7.5% for beverages). Foods that contain a significant amount of some of those nutrients per 100 g (or a defined portion) of the product can be considered as a “source of” that nutrient [21,28].

Although, currently, there is no official guidance about micronutrient supplementation in healthy individuals for the treatment or prevention of COVID-19 disease [29], European authorities have observed an increased in food supplements sold online with claims of a positive effect on the immune system or as effective in protecting against COVID-19 disease. We should remember that, till date, there are no authorized health claims for any substance as protecting against viral infection or improving immunity against any virus. Thus, consumers should be informed that those products are illegal and its intake could be harmful. With this objective, the European Commission (EC) have proposed a coordinated action plan on online offers and advertising of food related to COVID-19 disease [30].

### 3.2. Food as Source of Nutrients and Bioactive Compounds Involved in Immune System Stimulation

No single food contains all the beneficial compounds involved in the immune function improvement.

A varied and balanced diet could include all the compounds involved in these functions acting in a summative or synergistic way. Fruits and vegetables are the main sources of vitamin C and provitamin A, containing phenolic compounds and often prebiotic fructans. Nuts are sources of polyunsaturated fatty acids, B group vitamins, and minerals, such as Cu, Fe, Se, and Zn. Whole grains and legumes are sources of B group vitamins and minerals, such as Cu, Se, and Zn, as well as fiber, including oligo and polysaccharides, with prebiotic effect in some cases. Fatty fish are sources of polyunsaturated *n*-3 fatty acids, vitamin D, B group vitamins, and minerals, such as Fe and Se. Milk and dairy products, especially fermented products, are sources of vitamins A and D, as well as probiotic microorganisms. In addition, eggs and meat products are sources of vitamin A, vitamins D and B group, and minerals, such as Fe and Se [31,32].

Micronutrients have an essential role in the normal function of immune system with a positive effect on promoting health and nutritional well-being. Micronutrient intakes should come from a nutritionally balanced and diverse diet, including from fruits, vegetables and animal source foods. The intake of the mentioned micronutrients, in a varied and balanced way, fits perfectly into the concept of the Mediterranean Diet, which has traditionally been considered one of the healthiest dietary guidelines, according to different epidemiological studies [33,34]. In the context of the present situation, population should be aware of the importance of maintaining healthy eating habits to reduce susceptibility, as well as to reduce complications from COVID-19 [35]; however, it must be highlighted that an adequate intake of these nutrients is a positive contribution to the immune function, not a guarantee of immunity.

### 3.3. Scientific Evidence about Health Benefits from Food and Food Supplements, Related to Immune System and COVID-19 Disease

Scientific literature search carried out in the scientific database PubMed using the keywords “immune system” and “food”, provided 17,675 studies, with more than one thousand clinical trials in the last 10 years. When using the keywords “immune system” and “food supplement”, 3560 studies were found. From them, 788 reviews and 559 clinical trials were selected (Table 2), being the most relevant ones, 13 meta-analysis conducted in the last 5 years; and 19 systematic clinical trials and 2 meta-analysis (highest level of evidence) in the last year.

The information obtained in this search was unspecific as different disorders are related to the immune system, with low correlation with the objective of this work, the role of nutrients and bioactive compounds to improve immune system and fight against the COVID-19 disease.

A new scientific literature search was performed in PubMed considering human studies as inclusion criteria and the keywords “immune system”, “food”, “food supplements”, and “COVID-19”. When using the keywords “immune system”, “food”, and “COVID-19”, it was found a total of 141 studies, 74 reviews, 4 clinical trials, and 1 meta-analysis. When using “food supplement” as a keyword, only 44 studies were found, considering the most important ones, 29 reviews, 2 clinical trials/meta-analysis. In order to be more precise, a new search including the term “micronutrients” was performed, results are shown in Table 3.

The complex interaction between nutritional status and SARS-CoV-2 infection and disease can be explained by different mechanisms, those could include systemic inflammation, immune system impairment, sarcopenia, as well as other pre-existing associated diseases [36]. Most of the human clinical trials found in our search were mainly focused on the study of immunogenicity and safety of adenovirus type 5 COVID-19 vaccine, rather than on food effect.

Lu and collaborators performed an interesting systematic review and meta-analysis (including 28 studies) by comparing mortality-related risk factors of COVID-19, SARS, and MERS [37]. Authors concluded that advanced age, together with hypertension, diabetes, increased LDL cholesterol, blood urea nitrogen, and decreased albumin, among others, were factors associated with COVID-19 mortality. In addition, malnutrition and deficiency of micronutrients may negatively affect the COVID-19 recovery.

COVID-19 pathogenesis is quite complex and includes different processes as the suppression of host antiviral, innate immune response and oxidative stress induction. This is followed by a first stage of inflammation, which is responsible for the lung injury, tissue fibrosis, and pneumonia [38]. Thus, there are two ways to help fighting against COVID-19 disease, one with antiviral response through immune system boosting, and another with antioxidants with anti-inflammatory effect. In this way, nutritional therapy should be considered as important in patient’ care for COVID-19 survival, in addition to improve and shorter the recovery [39]. As an example, some micronutrients and bioactive compounds (such as vitamins C and E, carotenoids, and polyphenols) have exerted important anti-inflammatory effects and antioxidant properties, and can interact with transcription factors [40,41]. Different works have underlined the relevance of maintaining an optimal nutritional state to help the immune system against viral infections [1,42,43,44,45,46,47,48]. These authors make a special reference to micronutrients, as well as omega-3 fatty acids. They consider that all of them play an important and complementary role in supporting the immune system, so their deficiency could mean a decrease in resistance to infections and an increase in the disease burden.

Similar studies have focused on vitamin and mineral status of COVID-19 patients, analyzing the severity of symptoms and the evolution of the disease. Sometimes, the use of very high doses of some nutrients (vitamins and minerals) to fight against SARS-CoV-2 virus infection has been proposed. Even more, Sing (2020) considers that, in COVID-19 patients with non-communicable diseases, vitamins are epigenetic factors, which could improve immunity and reduce inflammatory response [49]. Galmes et al. (2020) studied the ten essential nutrients related to the immune system and the genetic factors that can limit their bioavailability. Results shown that intake levels of vitamins D, C, B_12_, and iron, have a positive health effect as they are correlated with lower COVID-19 incidence and mortality. This effect is more evident in populations with lower micronutrient status [50].

Some studies have focused on omega-3 polyunsaturated fatty acids, reported immunomodulation effects and an improvement of mood disorders, and thus may have the potential to enhance our immunity against COVID-19 with a positive mental impact [51].

Bioactive phytochemicals and immunomodulatory agents, such as polyphenols, with interesting properties, like reducing inflammation and preventing oxidation process [52], may also be interesting adjuvants in the treatment of SARS-CoV-2 infection helping to reduce the inflammation [53]. Different polyphenols have shown promising activity, such as glycyrrhizin or polyphenols (e.g., caffeic acid, kaempferol, resveratrol, curcumin, quercetin, catechin, or hesperidin) [54,55].

In addition to these nutrients, other bioactive components of food as microorganisms have shown positive effects on the body’s defense mechanisms. *Lactobacillus genus* strains, that take part of nasal microbiota, could protect from viral penetration and help the host’s immune system, and some traditional food are rich in lactobacilli strains [56].

Others have a positive impact on gut microbiota, even though our understanding on its mechanism is not complete [57]. Many lactic acid bacteria, such as lactobacilli and bifidobacteria, can compete with other pathogenic microorganisms in the colon, but also increase the immune response by different mechanisms [58]. These microorganisms are often conveyed in food, such as fermented milk (yogurt, kefir), and can reach and colonize the intestine, as probiotic microorganisms, enhancing these defense effects. Morais et al. (2020) discusses the relationship between the use of *Lactobacillus gasseri* and the proper intake of micronutrients and bioactive compounds from food, as a positive factor adjuvant nutritional therapy to stimulate the immune system function and decrease viral replication [59]. Take as an example, Shinde et al. (2020) studied the symbiotic combinations of probiotic bacteria, prebiotic dietary fiber and polyphenols, which can help regulate the immune response to restrain viral respiratory infections and temper the response of neutrophils, which can lead to acute respiratory distress syndrome [60].

Different authors reviewed the role of probiotics in the gut microbe modulation, gut homeostasis and improvement of gut barrier function. Authors reflect different positive effects as the production of interferon, the reduction of the respiratory infection symptoms and inflammatory response [61,62,63,64].

Related to specific food and its positive effects against COVID-19 disease, only one study was found and was related to propolis, which is widely consumed as a health aid and immune system booster. Results from pre-clinical studies suggest that propolis promotes immunoregulation of pro-inflammatory cytokines; thus, propolis can be considered as natural food supplement [65].

Besides food, nutrients and food bioactive compounds, researchers have focused on other biological compounds as melatonin, the main hormone of the pineal gland that influences many biological processes in the body. Melatonin levels decline considerably with aging, which is associated with several age-related diseases as oxidative damage and mitochondrial dysfunction. Studies focused on the relationship between melatonin and aging suggest an indirect antiviral effect [66] and the modulation of immune response and neuroinflammation caused by SARS-CoV-2 [67].

Now, taking into account the approved health claims related to the function of the immune system, we focused on the relevant reviews about the micronutrients associated with the immune system positive effects for patients with COVID-19, with vitamins D, C, zinc, and selenium being the most studied ones.

From all the micronutrients, Vitamin D is the one with more scientific evidence of positive effects against the COVID-19 disease as it is linked to a reduction of infection rates and an increase of improved outcomes in patients. Vitamin D, besides its activity as micronutrient, it is also known as an immunomodulatory hormone. Lately research has shown that vitamin D also influences immune cells and generally lowers inflammation.

Vitamin D stimulates the production of antimicrobial peptides, activates defensive cells, such as macrophages that could destroy SARS-CoV-2, and decrease the production of inflammatory cytokines which lead a prevention of cytokine storms [39,68]. In addition, vitamin D is an important regulator of the renin-angiotensin system (angiotensin converting enzyme 2, ACE2), that is exploited by SARS-CoV-2 to enter the host cells [41,48,68].

Mercola et al. (2020) reviewed the evidence that associates vitamin D with a reduction of the risk and severity of infections, including COVID-19 disease. This review includes the comparative analysis of 14 observational studies [69]. To help understanding the vitamin D antiviral mechanism, Siddiqui et al. (2020) reviewed the relation between serum vitamin D levels and viral infections, as vitamin D regulates the innate and adaptive immune systems [70]. According to Xu et al. (2020), vitamin D may play immunosuppressant effects, inhibiting cytokine release syndrome and suppressing important pro-inflammatory pathways [71]. This vitamin might avoid loss of neural sensation in COVID-19 by stimulating neurotrophins expression, and vitamin D may have a role in the induction of key neurotrophic factors.

Several epidemiological studies highlight vitamin D deficiency in the general population, either due to low sun exposure or a diet low in fat, being young children, elderly and obese people, the most susceptible population groups to experience this hypovitaminosis [72]. Multiple immune-related diseases are correlated with low serum levels of vitamin D [73]. This vitamin deficiency has also been detected in those affected by COVID-19 disease [74,75,76,77]. For this reason, the daily administration of 10,000 IU of vitamin D_3_ has been proposed to people at risk of suffering from this disease [78]. A controlled study is being carried out in these patients admitted to the ICU, therefore its efficacy is still unknown [79]. Considering the potential immunomodulatory role and antiviral effects of vitamin D, Isaia and Medico (2020) suggest that public health campaigns should be implemented to promote the consumption of vitamin D-rich food and explain the benefits of sunlight exposure, as well as to implement food supplements when needed, in populations with high prevalence of hypovitaminosis D [80]. All previous comments suggest a protective role of vitamin D in COVID-19 disease but many questions remain unanswered [81].

Vitamin C is also considered as a potential candidate to shorten disease course and reduce the severity of symptoms as it increases immunity due to its beneficial role as an immune booster and inherent antioxidant properties. Thus, it could increase the survival rate of COVID-19 patients reducing the hyper activation of the immune response [41,82]. Vitamin C prevents the onset of cytokine storm, which results in a decrease of viral yield [39,83]. These effects could be of interest in elderly people due to the immunosenescence process, to help COVID-19 vaccines in this population group. Holford et al. (2020) have reviewed Vitamin C positive effect as an adjuvant on respiratory infection, sepsis and COVID-19 treatment [84]. Authors give a recommendation of an oral supplementation in the range of 2–8 g/day to help attenuate the conversion to the critical phase of COVID-19 disease. Although Vitamin C has shown clear immunomodulatory effects, further clinical trials should be performed to prove the efficacy of vitamin C in treating COVID-19 patients [85].

Other micronutrients as vitamin E, together with the minerals zinc and selenium, are known to improve recovery from influenza viral infection and may be of interest in COVID-19 treatment, as Vitamin E is involved in the dendritic cells, influence interleukin IL-2 production, and T-cell regulation [41].

Selenium is a trace element crucial to human health with a wide range of protective functions. Selenium improves the function of cytotoxic effector cells, which is important to maintain T cell maturation and functions, as well as for T cell-dependent antibody synthesis [39]. According to Zhang et al. (2020), the intake of selenium over the nutritional requirements might suppress the life cycle and mutation to virulence of SARS-COV-2 while attenuating viral-induced oxidative stress, organ damage and the cytokine storm [86]. This positive effect could be of great interest in elderly people, those at particularly COVID-19 risk, as selenium deficiency is positively correlated with an increase in inflammatory cytokines [87].

Zinc is essential to maintain an adequate status of immune and redox systems, as well as natural tissue barriers (respiratory epithelium), which can contribute to avoid pathogen entry. In fact, its deficiency could be considered one remarkable risk factor that could predispose individuals to the COVID-19 infection and poor prognosis [88]. Zn salts appear to inhibit the replication of some viruses, including SARS-CoV. Thus, ensuring a proper intake of zinc might improve the host response and be protective against viral infections [89]. Pal et al. (2020) performed an interesting review on the Zinc and COVID-19 current clinical trials on going but have still not finished the results, although promising are not conclusive [90].

The following Table 4 includes a summary of main reviews previously commented.

To complete the results obtained in PubMed, an additional search has been carried out on the World Health Organization’s International Clinical Trials Registry Platform [24] and on the website ClinicalTrials.gov. This search resulted in a total of 190 clinical trials (81 trials related to the keywords ‘food supplement’ and ‘COVID-19′, 61 trials related to the keywords ‘vitamin’ and ‘COVID-19′ and 48 trials related to the keywords ‘micronutrients’ and ‘COVID-19′), as it is shown in Table 5; all of them ongoing studies.

The potential positive effects of food supplements, related to COVID-19 disease, can be achieved through either enhancing immune system, or specific chemical interactions of certain food compounds with host or virus targets, thus interfering with SARS-CoV-2 viral infective cycle [91,92,93,94].

Some promising clinical trials on the advantages of zinc supplementation in patients with COVID-19 have also been located and many of these studies combine zinc supplementation with vitamins D or C. These studies are aimed to find out whether ascorbic acid and zinc gluconate can decrease the duration of symptoms seen in patients with new diagnosis of COVID-19 or can prevent the progression of the severe manifestations of the disease, including development of dyspnea and acute respiratory distress syndrome, which may require hospitalization, mechanical ventilation, or can reduce the mortality.

Other clinical trials are aimed to investigate whether taking a dietary supplement of vitamin D, C, zinc, or a different kind of probiotics can reduce the risk of hospitalization, morbidity and/or mortality in participants diagnosed with COVID-19, and if these supplements can reduce the risk of infection with severe acute respiratory syndrome coronavirus (SARS-CoV-2). Although most of these studies are ongoing, correlations can be observed between the improvement of the disease prognosis in hospitalized patients by COVID-19 and the intake of supplements of certain vitamins and minerals.

Several studies are based on the knowledge that the vitamin D is synthetized by our own organism with the induction of sunlight that could contribute to the adaptive immunity and cellular differentiation, as well as maturation and proliferation of multiple immune cells. Furthermore, some researchers have detected that insufficient vitamin D concentrations were primarily responsible for bovine coronavirus infection in calves in the past. For this reason, they believe that vitamin D exerts an important immune modulator effect that could help preventing COVID-19 disease and, when infected, better outcomes in non-severe symptomatic patients.

There is only one clinical trial finished (NCT number: 04810949) conducted (during 6 months) in 41 patients with serum vitamin D levels ± 20 ng/mL. This is a comparative randomized clinical trial that evaluated the effect of 2 treatments in health personnel of Hospital Clínica Nova (Mexico): 1. Supplementation with 52,000 units of vitamin D3 monthly; 2. Hygienic-dietary measures: sun exposure 10 min a day plus recommendation of consumption of foods rich in vitamin D. The study completion date was 1 march 2021 but results have not been posted yet [95].

Although there are no definitive results yet, COVID-19 pandemic has provoked anxiety among worldwide population and fueled demand for potential cures and food supplements formulation with the different micronutrients previously mentioned [79,84,96,97,98]. The International Society for Immunonutrition [99] gives the advice to elderly people to increase the intake of nutrients, which have been shown to be effective in enhancing antibody immunity. It is the case of Vitamin E (134–800 mg/day), Zinc (30–220 mg/day), Vitamin C (200 mg–2 g/day), particularly for those people with low serum vitamin D status, Vitamin D (10–100 μg/day).

In any case, there is enough evidence to insist on the importance of maintaining a good nutritional status through the intake of a balanced diet, with an abundance of plant-based foods rich in micronutrients [100]. Along with specific food supplements, it could be a good strategy to help reducing the negative effects of COVID-19 [41,43,46,101,102,103].

## 4. Conclusions

The main tool to fight against COVID-19 disease is vaccination and vaccine efficacy can be optimized by ensuring nutritional adequacy of population, with special incidence in elderly people.

In addition, the supporting of immune system continues to be fundamental in the fight against infections, for acquiring immunity after vaccination, establishing the first line of defense after infection, and for fighting to overcome the disease if it develops. In the current situation of the COVID-19 pandemic, in addition to recommendations about correct hygienic practices in prevention of virus transmission, the intake of a varied and balanced diet, rich in micronutrients and bioactive compounds, should be recommended to improve our health status, and to keep our body in better conditions to deal with infectious agents.

Micronutrients play an important and synergistic role in supporting the immune system, so that their deficiency can reduce the resistance against infections. There are different Health Claims approved in the EU related to micronutrients (Vitamins B_6_, B_9_, B_12_, A, D, C, and Cu, Fe, Se) and the immune system support that can be used in food and food supplements.

Vitamins D, C, Zn, and Se have been thoroughly studied as a strategy to improve immune system helping to fight against the COVID-19 disease. To validate scientific evidence, different clinical trials are ongoing evaluating if the use of food supplements (including vitamins C, D, and Zn) can reduce the risk of infection with severe acute respiratory syndrome, or reduce the risk of hospitalization, morbidity, and/or mortality in participants diagnosed with COVID-19 disease. Preliminary results are promising but still not conclusive.

## Figures and Tables

**Table 1:** Nutrients with EFSA authorized Health Claim related to Immune system function. Based on: EU Register on nutrition and health claims made on food [20].

Food or Food Component	Conditions of Use *
*Health Claim: “Contributes to the normal function of the immune system” (Art. 13.1)* *Targeted population: General population*	
**Vitamin A**	120 µg/100 g (foods)
	60 µg/100 mL (beverages)
**Vitamin B_6_**	0.21 mg/100 g (foods)
	0.105 mg/100 mL (beverages)
**Vitamin B_9_ (folic acid and its derivatives)**	30 µg/100 g (foods)
	15 µg/100 mL (beverages)
**Vitamin B_12_**	0.375 µg/100 g (foods)
	0.1875 µg/100 mL (beverages)
**Vitamin D**	0.75 µg/100 g (foods)
	0.375 µg/100 mL (beverages)
**Copper**	0.15 mg/100 g (foods)
	0.075 mg/100 mL (beverages)
**Iron**	2.1 mg/100 g (foods)
	1.05 mg/100 mL (beverages)
**Selenium**	8.25 µg/100 g (foods)
	4.125 µg/100 mL (beverages)
**Zinc**	1.5 mg/100 g (foods)
	0.75 mg/100 mL (beverages)
*Health Claim: “Helps support the body’s immune system”, “needed as part of the body’s defenses”* (Art. 13.1)	
*Targeted population: General population*	
**Vitamin C**	12 mg/100 g (foods)
	6 mg/100 mL (beverages)
*Health Claim: “Contributes to maintain the normal function of the immune system during and after intense physical exercise” (Art. 13.1)* *Targeted population: General population*	
**Vitamin C**	200 mg/day
*Health Claim:“Contributes to the normal function of the immune system in children”* (Art. 14.1.b) Targeted population: Children (3–18 years of age)	
**Vitamin D**	0.75 µg/100 g (foods)
	0.375 µg/100 mL (beverages)

* Minimum threshold to apply claims according to Nutrient Reference Values, Regulation (EU) 1169/2011.

**Table 2:** Summary of the studies found in PubMed by using ‘immune system’, ‘food’, and ‘food supplements’ as keywords.

**Immune System—Food**		
Studies in the last 10 years	Studies in the last 5 years	Studies in the last year
Total: 17,675	Total: 9961	Total: 1505
3047 reviews	1844 reviews	360 reviews
1012 clinical trials	424 clinical trials	35 clinical trials
**Immune System—Food Supplements**		
Studies in the last 10 years	Studies in the last 5 years	Studies in the last year
Total: 3560	Total: 1802	Total: 236
788 reviews	433 reviews	68 reviews
559 clinical trials	222 clinical trials	15 clinical trials

**Table 3:** Summary of the studies found in PubMed by using ‘immune system’, ‘COVID-19′, ‘food’, or ‘food supplements’ as keywords.

**Immune System—Food—Covid-19**	**Immune System—Food Supplements—Covid-19**
Total: 238	Total: 70
128 reviews	45 reviews
6 clinical trials	2 clinical trials
2 meta-analysis	0 meta-analysis 0
**Immune System—Food—Micronutrients—Covid-19**	**Immune System—Food Supplements—Micronutrients—Covid-19**
Total: 59	Total: 33
45 reviews	23 reviews
1 systematic reviews	1 systematic reviews
0 clinical trials	0 clinical trials
0 meta-analysis	0 meta-analysis

**Table 4:** Main reviews found in PubMed by using ‘immune system’, ‘food’, ‘food supplements’, and ‘COVID-19′ as keywords.

Food/Nutrient/Bioactive Compound	Positive Suggested Role Against COVID-19	Reference
Micronutrients: vitamin and minerals	Support of the immune response	Fedele et al., 2021 [36]; Junaid et al., 2020 [45]; Pecora, et al., 2020 [46]; Singh, 2020 [49].
Vitamin C	Immunomodulatory and antioxidant action	Bae and Kim, 2020 [39]; Calder et al., 2020 [9]; Cerullo et al., 2020; Chen at al. 2020; Shakoor et al., 2021 [48].
Vitamin D	Immunomodulatory and antioxidant action	Bae and Kim, 2020 [39]; Biesalski, 2021 [74]; Calder et al., 2020 [9]; Cereda et al., 2021 [81]; Charoenngam et al., 2020 [73]; Isaia and Medico, 2020 [80]; Kumar et al., 2021 [68]; Laird et al. 2020 [72]; Mercola, et al., 2020 [69]; Shakoor et al., 2021 [48]; Siddiqui et al., 2020 [70]; Weir et al. 2020 [77]; Xu, 2020 [71].
Zn	Anti-inflammatory and antioxidant, reduces ROS in viral infections	Calder et al., 2020 [9]; Pal et al., 2020 [90]; Razzaque, 2020 [89]; Shakoor et al., 2021 [48]; Wessels et al., 2020 [88].
Se	Antioxidant role, ROS balance in inflammatory processes, immune-cell function	Bae and Kim, 2020 [39]; Calder et al., 2020 [9]; Shakoor et al., 2021 [48]; Zhang et al., 2020 [86].

**Table 5:** Main clinical trials found by using ‘food supplements’, ‘micronutrients’, ‘vitamin D’, and COVID-19 as keywords.

Clinical Trials
154 trials—keywords ‘food supplement’ and ‘COVID-19′
86 trials—keywords ‘micronutrients’ and ‘COVID-19′
68 trials—keywords ‘Vitamin D’ and ‘COVID-19′

Source: Clinical Trials Registry Platform [24].

## Data Availability

Not applicable.
